# Human brain local field potential recordings during a battery of multilingual cognitive and eye-tracking tasks

**DOI:** 10.1038/s41597-025-05222-2

**Published:** 2025-05-28

**Authors:** J. Cimbalnik, J. S. Garcia-Salinas, N. Hamedi, S. Prathapagiri, L. F. Sarmiento Rivera, M. Galanina, J. Dolezal, L. Jurkovicova, P. Daniel, M. Kojan, R. Roman, M. Pail, W. Sredniawa, W. Fortuna, M. Sluzewska-Niedzwiedz, K. Smarzewska, P. Reinacher, A. Weiser, T. Skok, T. Piernicki, A. Orzol, A. Lier, S. Seifzadeh, Bozena Kostek, Andrzej Czyzewski, Pawel Tabakow, Milan Brazdil, Michal T. Kucewicz

**Affiliations:** 1 Department of Biomedical Engineering, International Clinical Research Center, St. Anne’s University Hospital Brno, Brno, Czech Republic; 2 Brain & Mind Electrophysiology laboratory, BioTechMed Center, Multimedia Systems Department, Faculty of Electronics, Telecommunications and Informatics, Gdansk University of Technology, Gdansk, Poland; 3 Czech Institute of Informatics, Robotics and Cybernetics, Czech Technical University in Prague, Prague, Czech Republic; 4 Brno Epilepsy Center, Department of Neurology, St. Anne’s University Hospital, Faculty of Medicine, Masaryk University, member of ERN EpiCARE, Brno, Czech Republic; 5 Central European Institute of Technology, Masaryk University, Brno, Czech Republic; 6 Department of Neurosurgery, Wroclaw Medical University, Wroclaw, Poland; 7 Department of Neurology, Wroclaw Medical University, Wroclaw, Poland; 8 Department of Physiology & Biomedical Engineering, Mayo Clinic, Rochester, MN USA

**Keywords:** Cognitive neuroscience, Sensory processing

## Abstract

Intracranial human brain recordings from multiple implanted depth electrodes using stereo-EEG (sEEG) technology for seizure localization provide unique local field potential signals (LFP) sampled with standard macro- and special micro-electrode contacts. Over one hundred macro- and micro-contact LFP signals localized in particular brain regions were recorded from each sEEG monitoring case as patients engaged in an automated battery of verbal memory and non-verbal gaze movement tasks. Subject eye and vocal responses in both visual and auditory task versions were automatically detected in Polish, Czech, and Slovak languages with accurate timing of the correct and incorrect verbal responses using our web-based transcription tool. The behavioral events, LFP and pupillometric signals were synchronized and stored in a standard BIDS data structure with corresponding metadata. Each dataset contains recordings from at least one battery task performed over at least one day. The same set of 180 common nouns in the three languages was used across different battery tasks and recording days to enable the analysis of selective responses to specific word stimuli.

## Background & Summary

Invasive electrophysiological recordings from the human brain present a unique opportunity to study the neural mechanisms underlying cognitive functions^1–6^. One example of such a recording is the evaluation prior to surgical treatment of drug-resistant epilepsy when multiple electrodes are implanted in various brain regions to localize sources of seizure generation^7^. Stereo-EEG (sEEG) is one of the most popular techniques used for this purpose, which plans trajectories of every electrode lead implantation in a three-dimensional stereotactic frame of reference to record local field potential (LFP) signals from relevant cortical and subcortical areas while avoiding blood vessels and minimizing tissue damage. Compared to other deep brain recording and stimulation procedures used to treat movement or psychiatric disorders^8^, sEEG can employ a range of electrode types combining both standard macro-contacts and special micro-contacts to sample from a large scale of electrophysiological activities^7^. LFP signals recorded from the micro-contacts are generated by local neuronal assemblies, in contrast to the macro-contacts recordings from wider neural populations^9,10^. Micro-contacts can detect extracellular action potential from individual neurons, a.k.a single units, which were used to investigate cognitive functions related to memory, conscious awareness or even free will^1,11^.

LFP recordings are typically performed in particular cognitive tasks that are not repeated over time in any one subject. Chronic recordings from multiple tasks repeated within and beyond one experimental session are rare but necessary for robust and reproducible studies of neural activities over time. New implantable devices for deep-brain stimulation and recordings are now capable of continuous streaming of LFP and behavioral data from patients suffering from movement disorders, epilepsy or depression^12–14^. It is possible to record hundreds of sessions with a given cognitive task performed repeatedly over months and years^15^. These device technologies, however, are limited to the number of channels, different tasks, and patients that can realistically be recorded. A middle ground would be to perform multi-channel sEEG recordings during a battery of tasks repeated at least once in various groups of patients. In our previous dataset publication^16^, we recorded intracranial EEG of English speaking patients from multiple macro-contacts during performance of selected verbal and non-verbal cognitive tasks with the same set of word stimuli repeated at least once in two different behavioral paradigms. The behavioral gaze and auditory responses were manually annotated and pre-processed together with the eye-tracking signals.

Here, we compiled datasets of LFP recordings from macro- and micro-contact channels during a battery of repeated verbal and non-verbal eye-tracking tasks acquired in three languages from two different groups of epilepsy patients at Polish and Czech clinical sites. Polish patients were recruited at the Polish site while Czech and Slovak patients from the Czech site, which provides epilepsy treatment for both nationalities. Each patient performed the task in their native language. This is a major advancement in terms of robustness and reproducibility across different recording sites and cultural and linguistic backgrounds. The same pool of words translated from the original in English^17^ into Czech, Slovak and Polish languages was used repeatedly in three different behavioral paradigms, probing: free recall, paired-associate learning and individual word screening. Each common noun from the pool was encoded and recalled multiple times across the three paradigms in at least one experimental session. The dataset thus provides unique conditions for the analysis of, e.g., the ‘concept cells’ on the micro-scale of single unit LFP responses^18,19^, or specific memory traces on the macro- and/or micro-scale of ripple and other high-frequency oscillations^20–22^. These and other electrophysiological activities and behavioral responses can, therefore, be studied beyond one paradigm as particular stimuli are encoded and recalled in different memory tasks or maintained during distractor phases of these tasks or during other gaze-tracking tasks in the same or subsequent experimental sessions.

In summary, the datasets comprise multimodal signals: (1) large-scale electrophysiological LFP and single-unit recordings from over 100 macro- and micro-channels in any one patient, (2) vocalization and gaze tracking were used to limit body movements and acquire stable pupilometric and electrophysiological signals with no additional artifacts, (3) gaze position on the screen and pupil size to monitor subject’s focus of attention and provide a non-invasive biomarker of memory and cognitive states^23–25^. Each LFP signal has an assigned anatomical coordinates of its corresponding contact localization in a unified brain volume. Each auditory response in the verbal tasks has been semiautomatically transcribed in a particular language with the very onset and offset of word vocalization accurately marked in time - the source web-based transcription tool is freely available online (https://gitlab.com/brainandmindlab/medial-transcription-google). These are synchronized together with the eye-tracking signals and the behavioral events in the tasks, like the onset and offset of stimuli presented on the computer screen, and stored in a universal BIDS structure for rapid sharing and analysis^16^. Such intracranial and non-invasive multimodal recordings from cognitive performance are rare. A recently published dataset provides single unit activity, LFP and fMRI synchronized to watching particular scenes in a movie^26^. Here, the multimodal signals are recorded throughout multiple tasks with the same stimuli repeated within and beyond a single experimental session, making it possible to study the mechanisms of reactivation and initial consolidation of memory traces when word lists are repeated over subsequent sessions. This is the first step toward prolonged continuous monitoring of neural activities underlying memory and cognition over multiple tasks and experimental sessions.

## Methods

### Data collection

Data were collected from patients at two clinical institutions: St. Anne’s University Hospital, Masaryk University in Brno, and Uniwersytecki Szpital Kliniczny, Medical University of Wroclaw. Patients were of three spoken languages: Polish, Czech and Slovak.

Informed consent was obtained from each participant and all procedures performed in studies involving human participants were in accordance with the ethical standards of the corresponding institutional review board committees and their respective guidelines and regulations. Approvals obtained from St. Anne’s University Hospital (4 G/2022, Jan 12th, 2022) in Brno and Uniwersytecki Szpital Kliniczny (KB-831/2021, Oct 14th, 2021) together with the patient consent forms are available locally at each site upon demand.

**Fig. 1:**
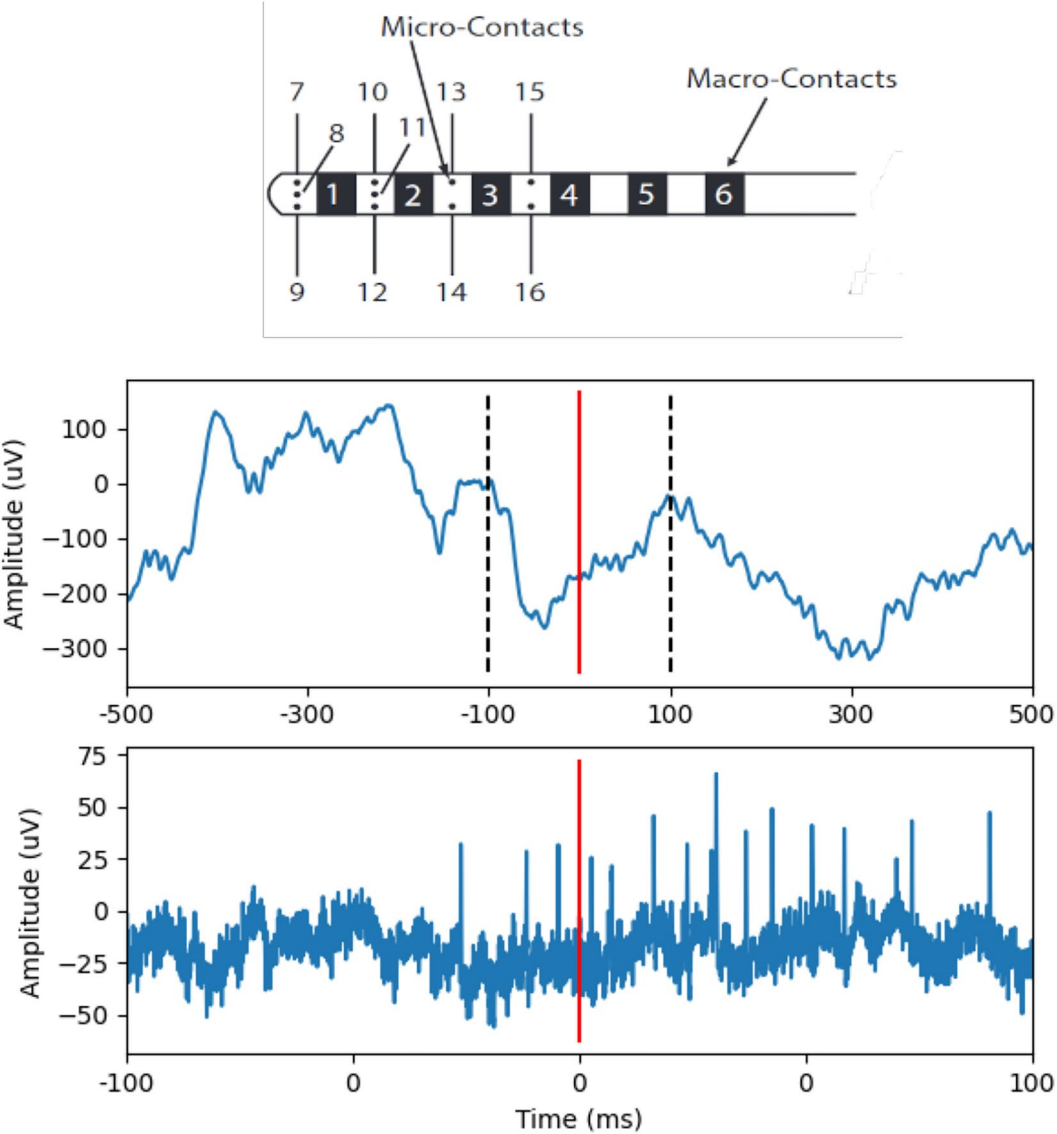
Hybrid electrode schematic (adapted from AdTech product catalogue) with examples of macro (top) and micro (bottom) contact acquired LFP signals. The hybrid electrodes used for signal acquisition in this dataset had multiple shaft microcontacts. The micro-contact signal span is represented in the macro-contact signal by black dashed lines. Notice multiple action potentials in the micro-LFP after the time of stimulus presentation (red line).

Electrophysiological signals were collected from electrodes implanted into the brain parenchyma with sampling frequency 5 kHz for St. Anne’s data (Easys2, M&I, Ltd., Prague, Czech Republic) and 4 kHz for macro contact and 32 kHz for micro contact Wroclaw data (Neuralynx, Digital Lynx SX, Bozman, MT, USA). The signals were collected from standard and hybrid clinical penetrating depth electrodes (AdTech Inc., Oak Creek, WI, USA and DIXI medical, Marchaux - Chaudefontaine, France) implanted into the brain parenchyma. The depth electrode contacts were separated by 5–10 mm spacing (Fig. 1). For each subject, the number and placement of the electrodes were determined by a clinical team with the goal of localizing epileptogenic brain regions as part of the patient’s evaluation for epilepsy surgery. The postimplantation localization of electrodes was done by co-registering the pre-implantation MRI to post-implantation CT using the Matlab Statistical Parametric Mapping software and subsequent manual marking. Following electrode implantation, subjects participated in a battery of up to five cognitive tasks, probing verbal memory and eye movements with tracking of pupil size and gaze position on the task display screen. All tasks were performed by the implanted patients resting comfortably in a hospital bed within one experimental task session a day. Task events were synchronized with the iEEG recordings using TTL pulses generated in the task computer and transmitted to a parallel input port for the iEEG signal amplifier. Behavioral responses (vocalizations of remembered word stimuli and eye movements on the screen) were processed after the experiment using microphone recordings and the pupillometric signals acquired on the task computer (i4tracking system, Medicton Group Ltd., Prague, Czech Republic). Task calibration, progression through the trials, and switching between the tasks were operated by the experimenter on the task computer without any need for patients to press the keyboard or make any motor movements except for the eye movements and the vocalizations. Patients were instructed to lay still in the bed, refrain from moving, and focus their gaze on the center of the screen. Only the task monitor was presented in front of the patients. The position of the monitor for optimal pupil detection was determined by visually checking real-time pupil detection before starting the battery and corrected throughout the session before initiating the next task by an automatic eye tracking calibration check to ensure high-quality pupillometry.

The battery of tasks started with a Smooth Pursuit (SP) task where subjects followed a horizontally moving dot in the middle of the screen with constant slow acceleration. SP was followed by a Free Recall (FR) task in which subjects were asked to remember 12 words presented one at a time on a computer screen and then to recall them in any order after a short distractor task with simple algebraic equations. After FR, another eye movement task was administered that included the paradigm of saccadic and anti-saccadic (AP) eye movements directed toward a target colored dot shifting position either to the right or to the left side of the screen center. Following the AP task was another verbal memory task that used 6 pairs of words displayed one after another for subsequent cued recall (in contrast to the list of 12 words presented for non-cued free recall in FR), following the same distractor delay as in FR, to probe paired-associate learning (PAL). For detailed descriptions of the aforementioned task please see our previous dataset work^16^. We also provide structured details about individual tasks in Table 1.

**Table 1:** Overview of individual tasks included in the dataset with interstimulus intervals and durations.

Task	Detailed description	Phases	Phase length (s)	Stimulus duration (s)	Interstimulus interval (s)	Repetitions
SP	Subjects are asked to follow a presented dot moving horizontally from one side of the screen to another at increasing velocity	Continuous	60	60	0	1
FR	Subjects are asked to remember lists of 12 words presented sequentially on the screen, followed by a short algebraic distractor task, and a period of freely recalling out loud the remembered words in any order	Countdown	5	1	0	15
		Encoding	30	1.5	1	
		Distractor	20	<20	0	
		Recall	30	30	0	
AP	Subjects are asked to follow a presented dot moving horizontally to either side of the screen (pro-saccade), or gaze to the opposite side (anti-saccade) according to color of the initial dot presentation	Continuous	380	2	1	1
PAL	Subjects are asked to remember 6 pairs of words presented sequentially on the screen, followed by a short algebraic distractor task, and a period of cued recalling out loud one of the remembered words from each pair	Countdown	5	1	0	15
		Encoding	30	4	1	
		Distractor	20	<20	0	
		Recall	30	4	1	
WS	Subjects are asked to watch the words presented on the screen. After certain period of time they are required to say out loud the last word they saw on the screen	Countdown	5	1	0	5
		Encoding	270	1	0.5	

The task session concluded with the word screening (WS) task where the patients were asked to focus on presented words and say the last presented word out loud when prompted by 3 question marks displayed on the screen. One trial of the task consisted of a presentation of 180 different words drawn from the same pool as in FR and PAL tasks. The subjects completed 5 trials in total therefore each word from the pool was presented 5 times. The WS task was designed to prompt a brain response to specific words, in order to identify anatomical areas that respond to specific concepts.

In summary, each task session comprised a battery of the five tasks administered in the following order: SP, FR, AP, PAL, WS. Depending on patients’ fatigue and willingness to continue the order was sometimes changed to: SP, FR, WS, AP, PAL. The aim was to record all tasks during one task session but this was not always possible due to limiting factors at the clinic or patient unwillingness to continue. The recordings were collected from a minimum of one daily task session with at least one task completed (Table 2).

**Table 2:** Overview of the total number of tasks and electrode recordings in individual patients.

Dataset	Language (ISO 639-1)	Subject	AP runs	FR runs	PAL runs	SP runs	WS runs	Micro contacts	Macro contacts
BR	SK	1	0	1	0	1	0	0	168
BR	CS	2	1	1	1	1	1	0	173
BR	CS	3	1	1	1	1	1	9	118
BR	CS	5	1	1	1	1	1	0	174
BR	SK	6	1	1	1	1	1	0	173
BR	CS	7	0	2	0	1	2	0	170
BR	SK	8	1	1	1	1	1	0	157
BR	SK	9	1	1	0	1	1	0	174
BR	CS	10	0	2	0	2	2	0	172
BR	SK	11	2	2	2	2	2	0	172
BR	CS	12	0	0	0	1	1	0	137
BR	CS	13	1	1	1	1	1	0	102
BR	CS	14	0	1	0	2	2	10	134
BR	CS	16	0	2	0	1	2	0	146
BR	SK	17	1	1	0	1	1	10	159
BR	SK	18	1	1	1	1	1	10	111
BR	CS	19	1	1	1	1	1	0	137
BR	CS	20	1	2	1	2	1	10	138
WR	PL	1	2	2	2	2	2	26	56
WR	PL	2	1	3	3	1	2	44	44
WR	PL	3	2	2	2	2	2	48	40
WR	PL	4	2	2	3	2	2	57	58
WR	PL	5	2	3	3	2	2	58	44
Total counts	CS - 11, SK - 7, PL - 5	23	22	34	24	31	32	282	2957

### Tracking of eye movements and pupil dilation

Recording of gaze position and pupil size was performed using a custom-made application developed from the i4tracking system (Medicton Group Inc., Prague, Czech Republic) designed for clinical use^27^. The recording was performed on a laptop computer connected to a 24-inch monitor screen with a resolution of 1680 × 1050 px, where the gaze position was tracked by high-resolution (2048 × 1088 px) and high-speed (up to 150 Hz sampling rate) external camera device placed right under the screen in front of the patient. Stimuli were displayed on the screen using a font size of 100 pt and were viewed from a distance of approx. 60 cm. Pupil position and size were detected by the camera device, corresponding to approx. 0.1 mm per pixel in the eye image. The camera device was positioned to capture the face area from forehead to the mouth. Two infrared light sources were emitted from the camera to capture the reflected light for pupil detection. Other sources of infrared light in the room were eliminated. Raw images from the camera were sampled at the rate of 150 Hz and were saved for extracting pupil size and position using a detection algorithm, which worked by fitting a general ellipse equation over the estimated pupil image. The pupil size in pixels was also converted to millimeters using measured interpupillary distance (IPD) and the IPD in the camera images. The reported pupil area was computed using the corresponding vertical and horizontal diameters in the ellipse area equation. Gaze position was determined by projecting the movement of the estimated center of the pupil onto the monitor screen area with the use of corneal reflection. Gazes outside of the screen area as well as the eye blinks were treated as missing samples.

Before the presentation of the task word lists, the eye tracker was calibrated for each recruited subject. In the calibration procedure subjects were asked to focus their gaze on nine points presented consecutively at specific positions across the diagonals and centers of the side edges of the display screen. Calibration was repeated before starting any new task in the session to ensure accurate estimate of the pupil size. Moreover, subjects were instructed not to move their heads and focus their gaze on the screen throughout all phases of the task trials. Despite these efforts, the quality of the eye-tracking was lower toward the end of each task due to slight shifts in the focus with changes in the head position. All stimuli were presented on the screen in a light gray color on a light gray background to minimize pupil responses to changing lighting and contrast. The testing was conducted in a room with low-light conditions that remained constant throughout the testing procedure.

### Localization of electrodes in the brain

Electrode localization was done using BrainVisa IntrAnat software v5.0^28^. The pre-implantation MRI scan was normalized to MNI space using Matlab (Mathworks Inc.) Statistical Parametric Mapping and the post-implantation CT was coregistered with it. The electrodes in the CT were manually marked and anatomical labels were assigned based on their MNI coordinates.

### Recording speech and adding annotations

Vocal responses of the subjects were recorded with an external condenser microphone. Speech was saved at a sample rate of 44100 Hz. Recordings were manually annotated after the experiments to mark the time and identity of the uttered words in custom audio editing software. This traditional approach of utilizing manual marking and labeling all vocalizations is laborious, time-consuming, and prone to human error, especially in case of large datasets with tens of task sessions performed.

To overcome these limitations we developed an automated transcription interface, which is compatible with the raw format of collected data. Our user-friendly interface is based on Whisper v3^29^, fine-tuned with the CommonVoice 17.0 dataset^30^ to ensure accurate transcription across various linguistic contexts, in our case English, Polish, Czech, and Slovak. Directories containing the audio files are scanned by the interface, which then proceeds to analyze and transcribe the recordings. After completing the transcription, all words are visually represented on the waveform, allowing to correct or delete any part of the transcription easily. Following modifications, the edited transcription can be saved in a convenient format for future preprocessing and analysis. Our approach has the potential to streamline data preprocessing and catalyze the process of labeling.

### Format conversion

**Fig. 2:**
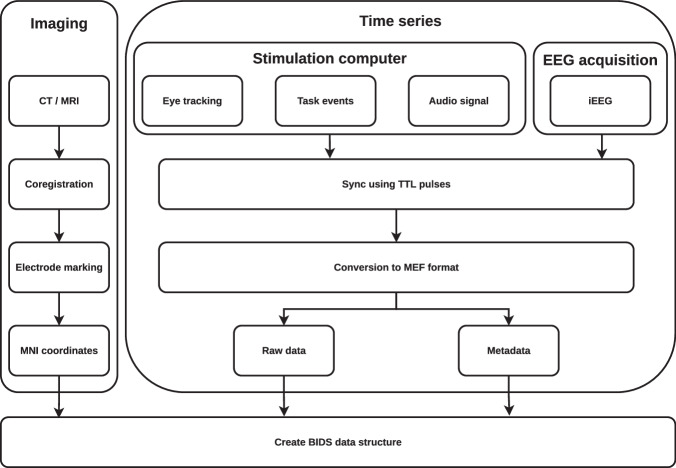
Flowchart of data conversion, annotation and electrode localization.

**Fig. 3:**
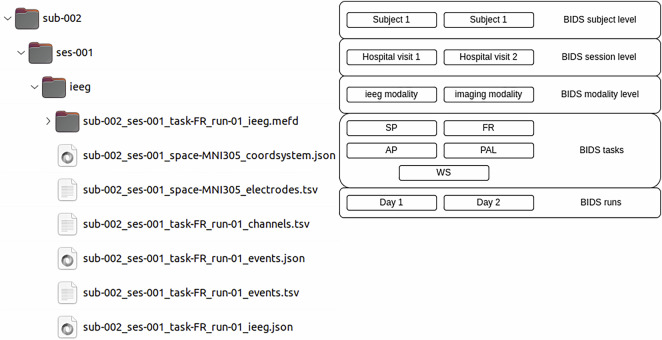
Example of the directory tree for one subject and task along with explanation of individual levels of the BIDS hierarchy.

The time series data (iEEG, i4tracking and audio) were processed by a software pipeline (Fig. 2), aligned in time based on TTL pulses that indicated specific annotated events in the tasks (Fig. 3), stored in Multiscale Electrophysiology Format (MEF) version 3^31^, and organized in BIDS data structure for further viewing and automated processing. All data were de-identified during the conversion process by omitting any information that could lead to the identification of the patient, such as name and date of birth.

## Data Records

The data is deposited at the EBRAINS repository^32^. Currently the data comprises recordings from 23 patients with 143 tasks performed. 15 patients (15) performed all the tasks. 11 patients performed at least one task in two runs. The number of patients that repeated individual tasks is: SP: 8, FR: 10, PAL: 6, AP: 5, WS: 10. A summary of the tasks performed with recordings from a specified number of implanted contacts is presented in Table 2.

The raw time series data (iEEG, audio, pupillometry) were stored in the MEF3 format^31^ due to its capability for handling data compression, variable sampling frequency across signals, and any signal discontinuities (Fig. 3). The MEF C library is publicly available at https://github.com/msel-source/meflib. The high-level API readers exist for Python (https://github.com/msel-source/pymef) and for Matlab (https://github.com/MaxvandenBoom/matmef). An overview of the processing structure is summarized in Fig. 2.

The Wroclaw and Brno datasets are stored in two separate folders and are organized in the BIDS data structure for iEEG^33^, which is supported by the International Neuroinformatics Coordinating Facility (https://www.incf.org/). The structure contains human-readable metadata and is widely used by the neuroscience community, enabling automatic processing by a wide range of available tools. The metadata are stored in.tsv (tab separated values) and.json files and contain information about the anatomical location of implanted electrodes (coordinates in MNI space), channel sampling frequencies, channel quality, and behavioral events (e.g. word presentations, recalled words, saccades). The structure of the data follows the BIDS standard version 1.2.1 with the folder levels arranged into a subject-session-iEEG-task-run order. The directory tree overview is depicted in Fig. 3 and individual file descriptions are provided in Table 3. The BIDS runs are individual performances of a particular task by one patient separated by 24–72 hours (i.e. task in separate task sessions).

**Table 3:** Description of all file types occurring in the dataset folder structure.

File	Description
dataset_description.json	Description of the dataset
README	Additional information for the dataset
participants.tsv	Information about individual subjects
participants.json	Contains information about columns in participants.tsv not specified in the BIDS standard.
sub-XXX_ses-XXX_space-MNI305_coordsystem.json	Description of the coordination system
sub-XXX_ses-XXX_space-MNI305_electrodes.tsv	Contains information about electrode and contact positions in MNI coordinates
sub-XXX_ses-XXX_space-MNI305_electrodes.json	Contains information about columns in electrodes.tsv not specified in BIDS standard
sub-XXX_ses-XXX_task-XX_run-XX_channels.tsv	Information about the channels in the particular task (sampling frequency, filters, reference, etc.)
sub-XXX_ses-XXX_task-XX_run-XX_events.json	Description of individual events in events.tsv
sub-XXX_ses-XXX_task-XX_run-XX_events.tsv	Events and times that happened during the recording (encode, recall, saccades, etc.)
sub-XXX_ses-XXX_task-XX_run-XX_ieeg.json	Description of the ieeg recording (task information, number and kind of recorded channels, etc.)
sub-XXX_ses-XXX_task-XX_run-XX_ieeg.mefd	Raw iEEG data in MEF3 format folder
sub-XXX_ses-XXX_task-XX_run-XX_ieeg.rdat	MEF3 format record data file
sub-XXX_ses-XXX_task-XX_run-XX_ieeg.ridx	MEF3 format record indices file
XXX.timd	MEF3 format time series folder
XXX-XXXXXX.segd	MEF3 format segment folder
XXX-XXXXXX.tdat	MEF3 format time series data file
XXX-XXXXXX.tidx	MEF3 format time series indices file
XXX-XXXXXX.tmet	MEF3 format time series metadata file

The root dataset folder contains readme and dataset description files with general information about the dataset and participants.tsv describing the metadata of individual subjects. The “code” folder contains an ipython notebook with scripts to guide the users and provide easy access to the data. The BIDS subject and session levels of the dataset do not contain any information. The BIDS session level was implemented in case the same patients are reimplanted in the future. The ieeg level contains multiple files and folders directly related to individual recordings (see Table 3). Each run represents one performance of a particular task. The events in _events.tsv without the trial_type information are the original ttl events as sent by the stimulation laptop. In FR, PAL and WS the events with trial_type information provide detailed description with specific words during encoding and recall along with distractor phase events. In SP the events represent the change in moving dot direction. In AP the events show information about fixation and the following anti/pro-saccade event. The.json files provide general descriptions of the recordings and descriptions of the columns in.tsv files that are not specified in the BIDS standard.

The dataset is available under EBRAINS Data Use Agreement via the EBRAINS Knowledge Graph^16^.

## Technical Validation

Compared to the previous dataset publication^16^, where we manually inspected and subjectively identified channels with noisy or corrupt LFP signals coming from contacts that were damaged or outside of the brain tissue, we applied custom-made automated tools like^34^ for any specific validation analysis. Likewise, a semiautomated procedure was applied to mark the times and annotate correct or incorrect recall of words in the audio signals of the patient responses. Our goal was to provide objective pre-processing of the signal with manual supervision of outcomes from the automated analyses. Accurate synchronization and alignment of the electrophysiological, pupillometric,audio and single unit signals with the task events was validated in the following examples of memory and cognitive effects found in these signals.

**Fig. 4:**
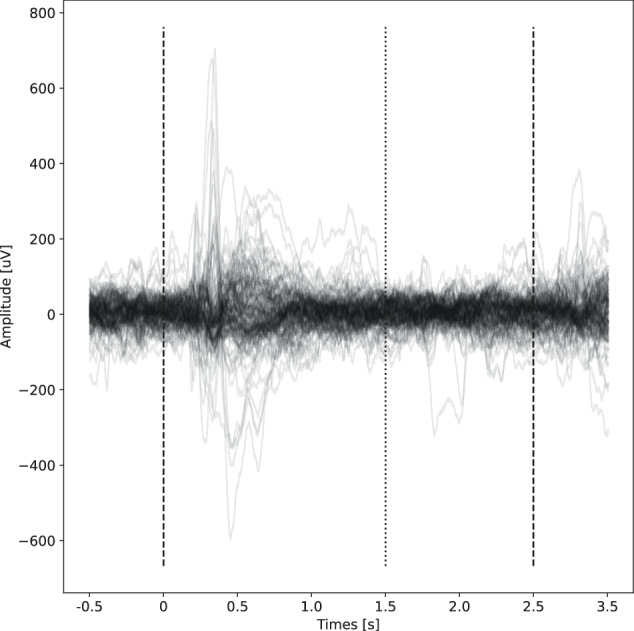
Example of aligned event related potentials for St. Anne’s patient 6, FR task. Each black line represents an averaged signal from one channel. The dashed vertical lines show the time of word presentations, while the dotted line represents the time when the word disappears from the screen.

We first carried out a simple analysis by plotting averaged signals from each task aligned with the stimulus in each task, which were defined as follows: SP - change of dot direction on the screen, AP - change of dot colors on the screen, FR; PAL; WS - presentation of a word/s on the screen. We then visually verified the alignment of event related potentials (ERP) with the stimulus time (Fig. 4). We provide a simple jupyter notebook for ERP calculation in the “code” directory within the dataset.

We plotted changes in the pupil size induced by encoding and free recall of words memorized in the FR task. Data were collected from seven participants, each completing a single session of the FR task. The task involved presenting 15 lists of 12 words on a computer screen, followed by a recall phase where participants attempted to remember as many words as possible. For each list, trials were categorized based on the number of words recalled. If a participant recalled 5 or more words, the recalled words and their corresponding encoded words were classified as “good” memory trials. Conversely, trials where 4 or fewer words were recalled were classified as “poor” memory trials. Lists where no words were recalled were excluded from the analysis, as these instances likely reflected missed attention rather than memory performance.

The pupil size was analyzed around the moment of word presentation for memory encoding and the moment of vocalization for memory recall. A two-way analysis of variance (ANOVA) was conducted to compare the mean pupil area across all good (n = 203) and poor (n = 262) trials for each 10 ms time bin (Fig. 4) to test the effect of the trial type (1 d.f.) and the subject (6 d.f.) on the normalized pupil size.

Pupil size was estimated by fitting an ellipse over the eye image, providing precision beyond a single pixel, corresponding to approximately 0.1 mm. The reported pupil area was derived as an average from measurements of both the left and right eyes, utilizing their respective vertical and horizontal diameters in the ellipse area equation. To ensure continuous signals, missing samples were filled through linear interpolation between neighboring samples. The total blinking time per subject was less than 5% of the entire recording duration.

**Fig. 5:**
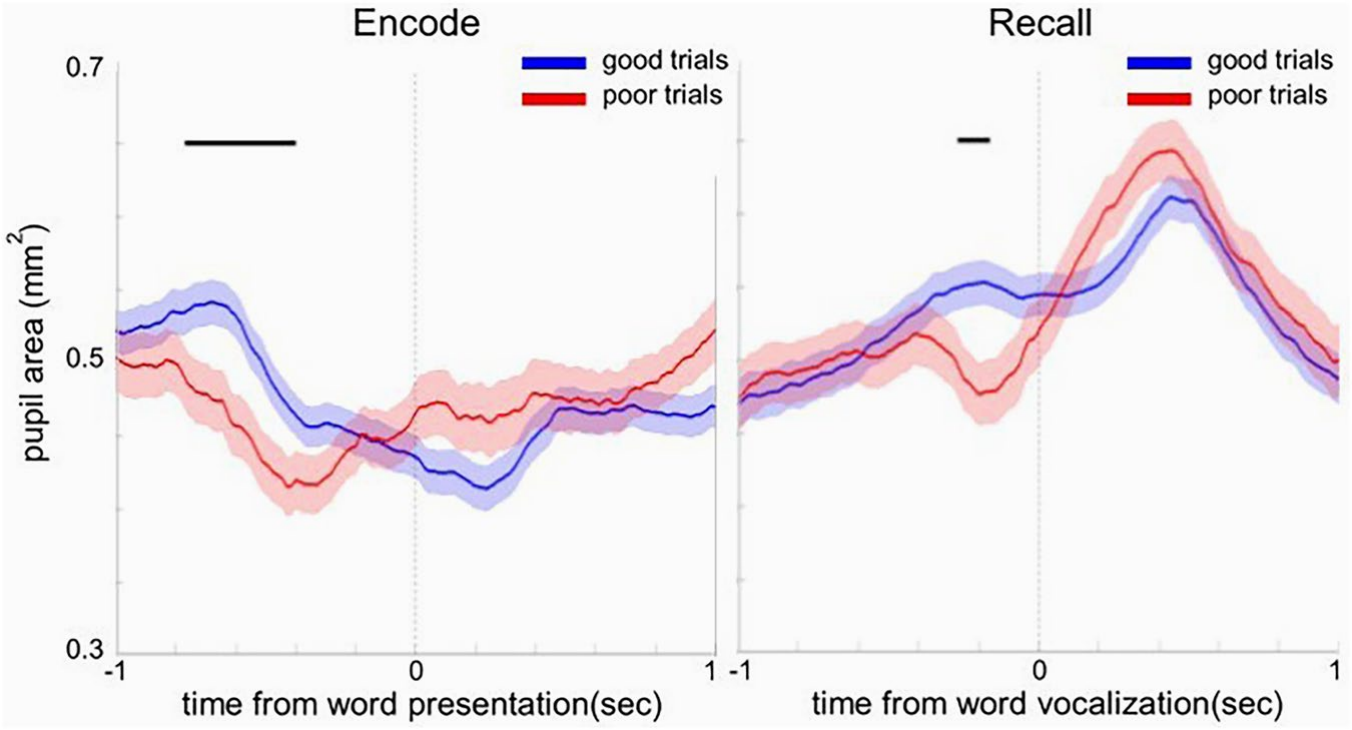
Pupil responses around word presentation and verbalization differentiate between good and poor memory trials. The diagram on the left shows subject-averaged pupil size aligned to the moment of word presentation, while the diagram on the right shows subject-averaged pupil size aligned to the beginning of word recall. Good trials (>4 words recalled) are depicted in blue, and poor trials (<5 words recalled) in red. The shaded regions indicate the standard error of the mean, and the black horizontal bars mark the time periods during which a significant effect of the trial type was observed in a given time bin (ANOVA, 1 d.f., p < 0.05). Notice the opposite pattern of pupil constriction and dilation, respectively, induced by memory encoding and recall.

The validation analysis confirmed our previously reported patterns induced by memory encoding and recall of pupil constriction and dilation, respectively^25^. These patterns were more pronounced for the good memory trials. Notably, significant differences between the good and poor trials were observed 250 milliseconds around the time of word presentation for the encoding, and up to 500 milliseconds before word verbalization for the recall (Fig. 5).

From the same subject data we then extracted epochs of the pupil size and LFP signals from a total of 401 recalled word trials. During this phase of the FR task, participants recalled as many of the 12 words presented during the encoding phase as they remembered in any order. We observed a correlation between the mean count of individual High-Frequency Oscillations (HFOs) detected in all LFP channels and the mean pupil area around the start of word vocalization.

Each HFO burst was detected using a Hilbert transform-based method^35,36^ in a broad range of 50–500 Hz of the frequency spectrum. The signal was first filtered in a series of logarithmically spaced frequency bands and each filtered signal was transformed to its pwoer envelope using Hilbert transform and z-scored. The signals were thresholded with mean + 3*std for putative detection of HFO. The detections overlapping in time and in adjacent frequency bands were merged into one putative HFO detection. The peak HFO frequency was determined at the highest fraction of std above the mean within the putative detection and the first onset and last offset were marked as detection start and stop. As the final step, each putative HFO was thresholded by a threshold of the time span of three oscillations at the peak HFO frequency. Putative detections shorter than this threshold were discarded.

**Fig. 6:**
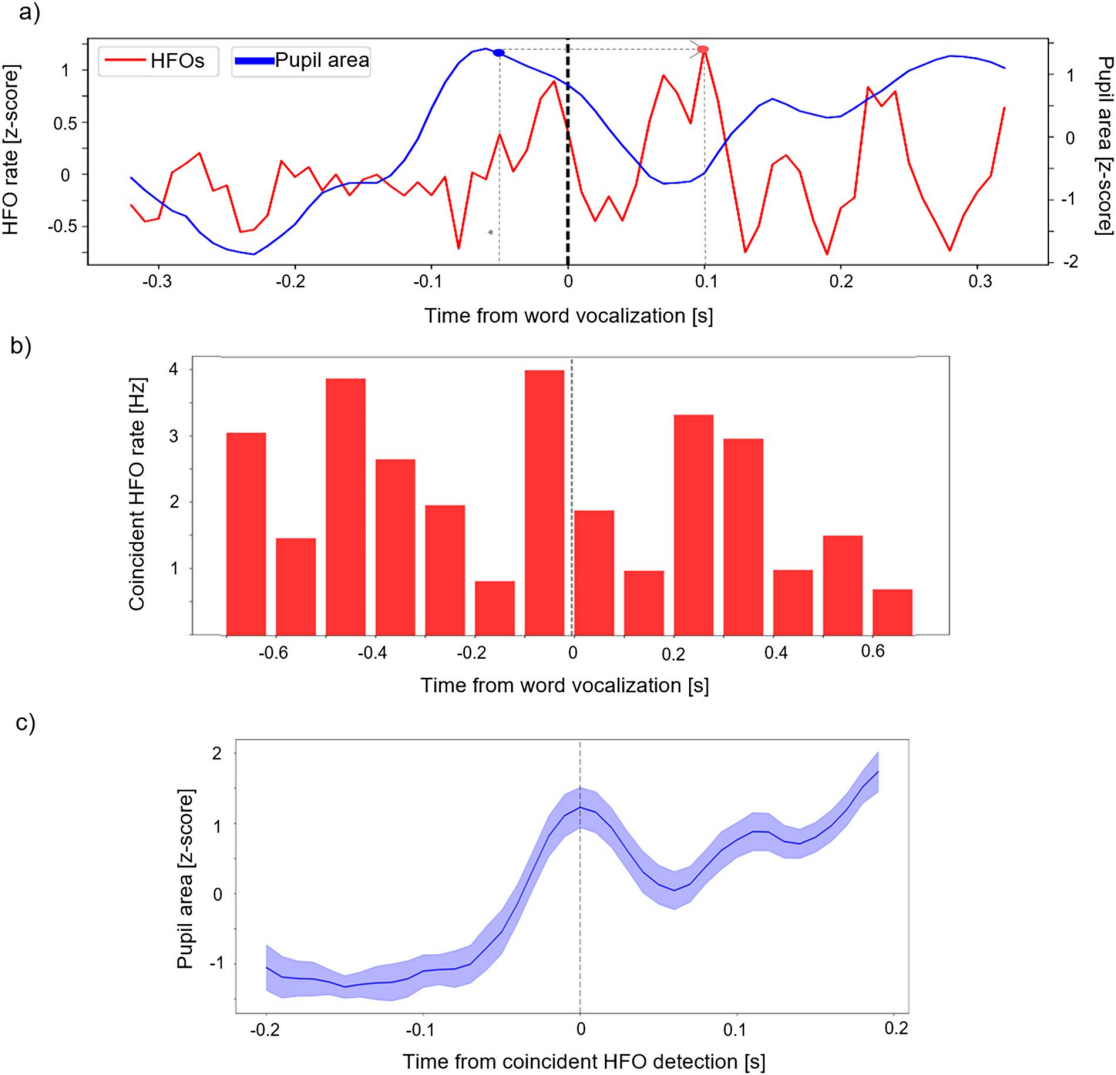
Memory recall induces an correlated increase in HFO detections and pupil size. (**a**) The recall showed pupil dilation before word vocalization and an increase on the HFO rates around the vocalization, significant Granger causality (* for p-value < 0.05) was found between the pupil dilation and increase of average HFO rates at 150 ms. (**b**) Coincident HFO detection events between the LFP channels peak around the same time of recalled word vocalization. Notice that this synchronous pattern of global activation is peaking 100 ms before the onset of verbalization, suggesting involvement in the memory retrieval processes. c) Pupil size around the time of the coincident HFO detection reveals progressive dilation, which directly associates the pupillometric and the electrophysiological activities.

The pupillometric and electrophysiological activities from all electrodes of 9 subjects (N = 9) were aligned together, increasing and leading up to the recall of memorized words (Fig. 6a). The pupil signals were prepared as described above with a moving average applied to smooth the signal.

Coincident instances of HFOs across all LFP channels were detected by applying convolution across temporal (time bins) and spatial (electrode channel) dimensions (5 × 5 mean kernel) to identify elevated HFO coincidence rates across the task phase and brain regions. A thresholding step was applied to filter out less significant HFO coincidences, activity higher than the mean + 2 standard deviations, involving fewer channels to remove noise or artifacts from the analysis. As expected, these coincidence events peaked immediately before the beginning of the recall vocalization (Fig. 6b), corresponding to the peak in the overall HFO rate (Fig. 6a).

Finally, we found that the pupil dilation was directly associated with the coincident HFO events. At the moment of coincident HFO detection, the pupil size exhibited a progressive dilation before the event onset (Fig. 6c). This dilation pattern around memory recall is congruent with the general dilation pattern observed in the recall phase of the task^25^, as plotted in the previous figure.

**Fig. 7:**
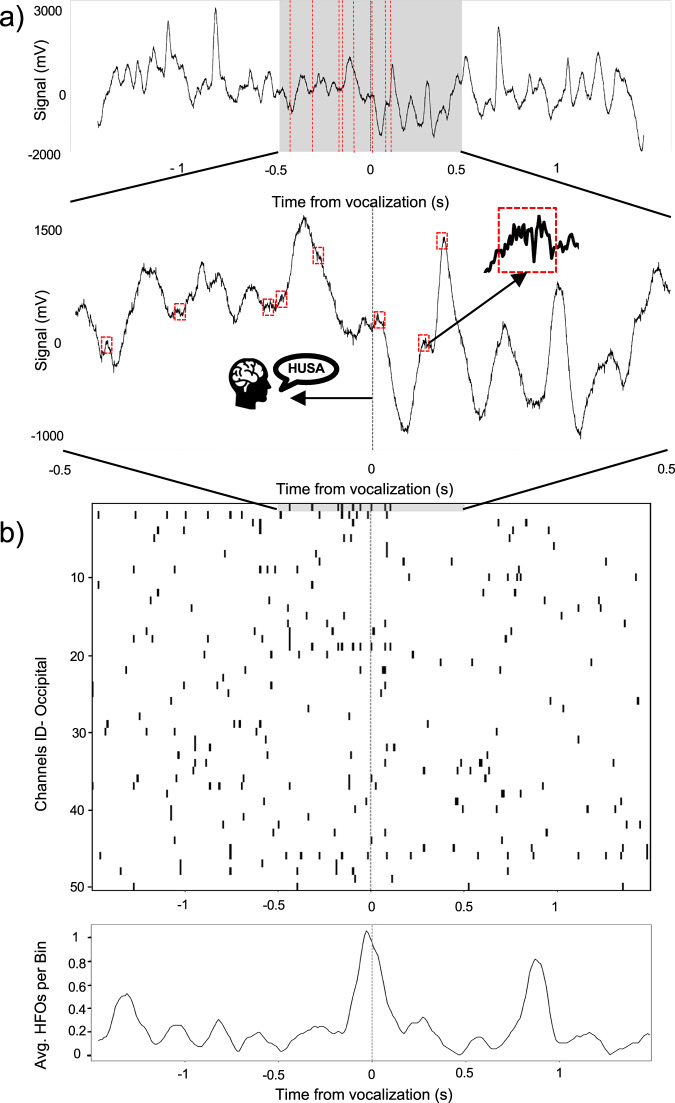
Temporal coordination of HFO bursts is associated with memory recall. (**a**) Raw LFP signal from an example channel in the occipital cortex reveals increased detection rate of individual HFO bursts right before beginning of verbalizing a freely recalled word. (**b**) (top) Raster plots summarize HFO bursts as point processes detected in 10 ms bins around the moment of recall across all occipital channels in this example patient. Notice that the increasing rate of HFO bursting leading up to the free recall is coordinated in time between multiple channels in the absence of any visual stimulation. (bottom) Summary of the increased HFO bursting across the occipital channels shows a peak of coordinated activity preceding recall verbalization.

The observed patterns of synchronized HFOs in the LFP signals leading up to word recall along with the correlated pupil dilation likely reflect cognitive processes required for retrieving memories. These temporally coordinated HFO events were apparent even in the visual areas of the occipital cortex, where we detected discrete bursts of oscillations in the raw LFP signals (Fig. 7a) between 50–500 Hz using methodology from our previous work^35,36^ and treated them as point processes at the time of spectral power peaks in the signal^37^.

Here, we plotted all HFO bursts detected on channels implanted in the visual cortex during the free recall of the Czech word “HUSA” (Fig. 7b). The resultant raster plot shows an increased rate of HFO detections preceding the moment of vocalization on multiple channels. This timing corresponds with the memory retrieval processes engaged right before the recalled word is uttered. It is worth noticing that this occipital cortical activation around recall occurs in the absence of any visual stimuli presented to the subjects, presumably suggesting engagement of mental imagery of the recalled words.

**Fig. 8:**
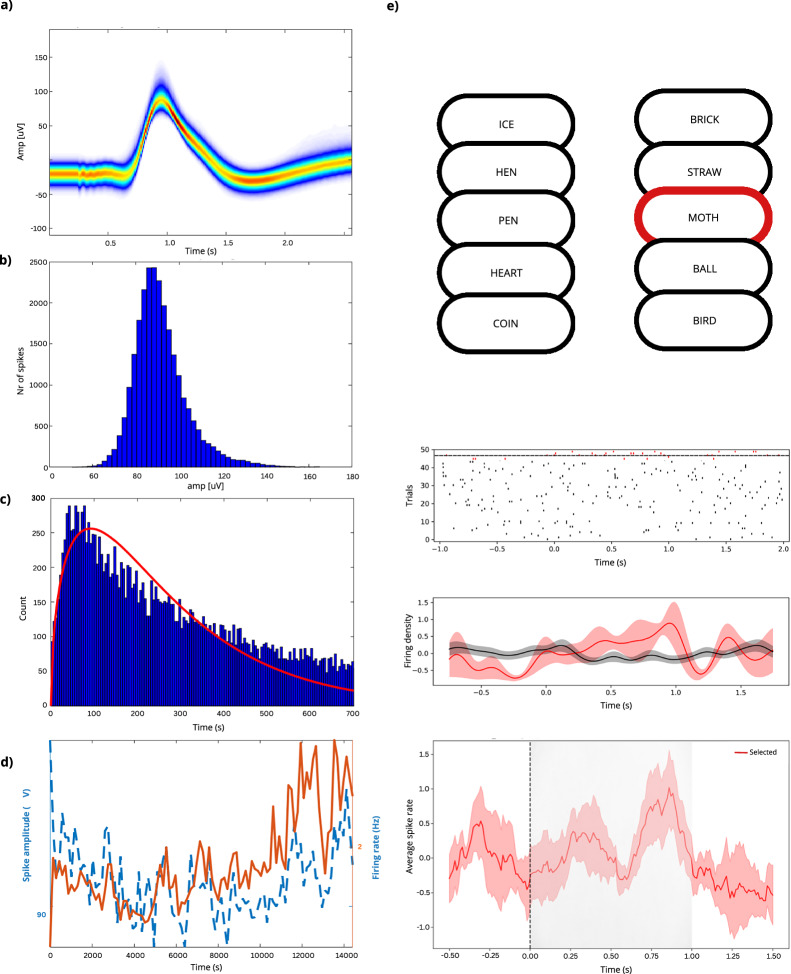
SUA is modulated by cognitive performance in the battery of verbal and gaze-tracking tasks where neurons respond to specific words. (**a**) Cumulative density plot of all overlaid action potential waveforms (n = 9543) confirms the highest density (warm colors) around the average amplitude values. (**c**) Histogram of the inter-spike intervals (ISI; each bin is 1 ms) reveals an irregular firing pattern with most spikes fired at ISI >3 ms (beyond the refractory period) and <200 ms, reflecting moderate bursting without a single predominant rhythm (red line is the smoothed average). (**d**) Summary of all spikes detected throughout the entire recording session shows a stable firing rate (orange line) and spike amplitude (blue line) across time. (**e**) Neurons responding to specific words increase their firing rate after the onset and peak just before the offset of the preferred words. **The Word Screening Task consists of five trials, where 180 words from the pool are presented in five blocks - once per block. Word presentation within each block follows the same order;** the left upper panel shows an example of ten words. Raster and the line plot below summarize spiking rate of the neuron shown in a–d in response to five presentations of preferred (red) and non-preferred (black) words. Summary of all spiking responses to preferred stimuli (N = 4 patients, n = 139 cells) confirms elevated firing rates in anticipation of the next word presentation and during encoding of preferred words displayed on the computer screen (gray area).

We validated the single unit activity (SUA) analyzed from the micro-contact LFP signals recording extracellular action potentials, also known as spikes, from individual neurons. Spikes were clustered and isolated into SUA throughout the entire recording session using the software Osort^38^, revealing modulation in the firing rate between the memory and gaze-tracking tasks. Here we show an example unit, firing on average 2–3 spikes per second with consistent waveforms, action potential amplitude, and inter-spike interval (Fig. 8a–c). In total, there were 9543 spikes isolated to this single unit recorded in the hippocampus with less than 0.56% of them falling into the refractory period. This unit showed an elevated firing rate during the verbal tasks (Fig. 8d,e), suggesting its involvement in the cognitive processes engaged in these tasks.

## Usage Notes

Libraries pybids^39^ or bids-matlab (https://github.com/bids-standard/bids-matlab) along with the libraries for MEF3 reading mentioned in the data records section can be used to programmatically navigate through individual tasks and subjects and to load the data for processing. The folder “code” in the dataset root directory contains a jupyter notebook with example code for reading the data in Python. The signals are not preprocessed intentionally to provide the data in a raw form and allow the users to perform their own preprocessing. The iEEG signals originating from epileptogenic tissue are not excluded but the pathological activity can be detected using open-source tools such as EPYCOM library (https://gitlab.com/bbeer_group/development/epycom/epycom). The pupillometry signals contain blinking artifacts and artifacts caused by clinical settings such as movement due to discomfort or pain. The pupillometry signal artifacts can be eliminated by using published methods^40^. The data stored in MEF can be viewed using PySigView^41^, a custom-made python viewer, or the SignalPlant program^42^. We will provide support to the users if they encounter any problems using the aforementioned methods.

### Limitations

The data set does not include the brain images for the sake of protecting patient privacy^43^.

## Supplementary information

Word pool table

## Data Availability

To provide an easy way to detect and analyze electrophysiological activity in iEEG signals we make our codes available at GitLab (https://gitlab.com/brainandmindlab/memory_encoding). Currently, the repository contains a python script to automatically process the individual BIDS layers of the dataset (subject, task, run, channel) using the EPYCOM library^44^. The library is focused on iEEG processing and contains a set of algorithms for automated detection of high-frequency oscillations^36,45,46^, interictal epileptiform discharges^47^ and for computation of univariate and bivariate features^48,49^. The repository will be gradually updated with scripts for statistical analyses and result visualizations.
